# RACIAL-ETHNIC DISPARITY IN DENTAL CARE IN NURSING HOMES

**DOI:** 10.1093/geroni/igz038.920

**Published:** 2019-11-08

**Authors:** Zhiqiu Ye, Bei Wu

**Affiliations:** 1 University of Rochester Medical Center, Rochester, New York, United States; 2 New York University, New York, New York, United States

## Abstract

Minority older adults are at higher risks of poor oral health. Little is known about the extent of and the contributing factors to racial/ethnic disparity in dental care quality in the long-term care settings. Previous studies suggest that organizational and system-level factors are key determinants of oral health among minority older adults. We examined the racial/ethnic disparity in dental care delivery in nursing homes (NHs) by facility and market characteristics. We analyzed the 2000-2016 national Inspection Survey data for all certified-NHs (n=248,975 facility-years). Two designated deficiency citations were used to measure dental care performance. Generalized estimating equations were used to compare the rates of deficiency citations among NHs in different quartiles of the share of minority residents, adjusting for facility characteristics, market characteristics, year and state fixed effects. Overall, compared to NHs in the lowest quartile of the share of minority residents (average % minority residents =0.24%), NHs in the highest quartile of the share of minority residents (average % minority residents = 46.5%) and those in the second highest share (average % minority residents=13.9%) had 46.8% and 31.2% higher odds of receiving dental care citations(p<0.001 for both), respectively. The increased citation rates persisted over time (p=0.40) and were greater among for-profit NHs (p=0.02). Our study suggests that minority older adults in NHs are disproportionately affected by poorer dental care performance. There is a great need to improve quality of dental care in NHs, particularly for those that are for-profit and those that disproportionately serve minority residents.

